# Mobile cognitive testing: opportunities for aging and neurodegeneration research in low- and middle-income countries

**DOI:** 10.7189/jogh.09.020313

**Published:** 2019-12

**Authors:** Graciela Muniz-Terrera, Tam Watermeyer, Samuel Danso, Craig Ritchie

**Affiliations:** 1 Edinburgh Dementia Prevention, Centre for Clinical Brain Sciences, University of Edinburgh, Edinburgh, Scotland, UK; 2 Global Prevention of Dementia Programme (GloDePP), Centre for Global Health Research, University of Edinburgh, UK

## LESSONS LEARNT AND CHALLENGES OF TRADITIONAL DESIGNS AND TESTING MODES

In all settings and communities, studies of ageing, brain health and neurodegeneration benefit from the accurate measurement of cognitive function to detect within person change, which is important for the identification of individuals at higher risk of developing neurodegenerative disease. The detection of within person change necessitates longitudinal designs where individuals’ cognitive functions are assessed repeatedly over time [1] with sensitive measures from the earliest stages of neurodegeneration [2]. The number of follow up occasions in these designs has repercussions for the ability to accurately estimate within person change. For example, studies with only 2 assessments per person are of limited use for within person change calculations as phenomena such as regression to the mean and the “horse racing” effect [3] would affect estimations, studies with 3 assessments only allow for the estimation of linear change whereas the reliable estimation of non-constant rate of change requires at least 4 measurement occasions [4].

In addition, extensive literature shows how dropout and death are highly prevalent in longitudinal studies of older adults, which are likely to be more marked in studies with a long follow up. Missing data are possibly informative in studies of older adults [5], with very healthy individuals or individuals in very poor health being more likely to dropout or die during the study follow up [6]. Various factors have been identified as predictors of dropout in longitudinal studies of ageing [7,8] and multiple strategies developed to minimize dropout and maximize retention and engagement of study participants [7-9].

A large body of evidence has also identified differences in research engagement across and within populations. For instance, American ethnic minorities individuals, similarly to Aboriginal individuals in Australia are less inclined to join research studies than white Americans and white Australians respectively [10,11].

How these and other factors known to be associated with dropout, retention and engagement in research studies in rich economies operate in LMICs is an area where further research is required.

In most existing longitudinal studies, data collection waves are scheduled at pre-determined times. Yet, despite researchers’ intentions, the timing of these collections are usually beyond the strict control of the investigators. Factors affecting the timing of data wave collections often depend on practical issues such as the securement of funding and having access to teams to implement the data collection. Moreover, because of the important human and financial resources required for these efforts, assessments are scheduled at occasions that are far apart.

The timing and separation of data collection waves is highly relevant as they impact the researcher’s ability to make certain critical inferences. In existing longitudinal studies, the time elapsed between data collection waves vary from 6 months to several years [12]. When a prolonged time interval elapses between data collection waves, not only dropout and death are more likely to be more pronounced, but chances of critical events occurring between testing occasions increase and opportunities to detect them and evaluate their impact are more likely to be missed. For example, it is possible that individuals experience catastrophic health events such a stroke in between study waves, and hence, the opportunity to understand the impact of such events on function may be missed. In sum, although traditional longitudinal designs have provided good opportunities to understand change in function in studies associated with ageing, they also present researchers with multiple challenges that may be exacerbated when similar designs are implemented in LMICs contexts.

Independent of the design of the studies, the measurement of cognitive function in studies of older adults is often made in the form of self-reports, reports by proxies, or by performance-based tests. Whilst self-reports and reports by proxies can be affected by retrospective reporting biases and other factors [13], performance-based tests are more objective measures of function. Yet, their routine use in longitudinal research studies is not without challenges. To begin with, psychometric properties of the tests may result in biased estimates of within person change, as some tests may be less sensitive to change in individuals who are at ceiling or perform very poorly. For instance, similarly to the Mini Mental State Exam [14], the most widely used test to measure global cognitive function, several other cognitive tests commonly used in studies of ageing and neurodegeneration are also known to have non-standard distributions. Moreover, tests optimized for use in older people with dementia have little or no value to detect the subtle expression of underlying neurodegeneration in younger people with the earliest changes in their brains.

Likewise, some cognitive tests are known to be biased towards some subgroups. For instance, the MMSE is known to be biased against individuals with sensory impairments, and as a result, it may not be the optimal tool to measure cognition in these individuals [15].

Taken together, although well established and traditional testing batteries are safe choices for testing participants in LMICs and also provide opportunities for direct comparison of results across studies and countries, the adoption of new testing batteries and implementation of innovative data collection means present interesting opportunities to advance knowledge in these populations. In essence, the learnings made in research uniquely conducted in wealthier countries should influence design of new research in LMICs which are not burdened by the legacy issues that sometimes acts against innovation in more traditional research settings. This could mean that psychometric test innovations could be first applied at scale in LIMCS from which other parts of the world can learn.

**Figure:**
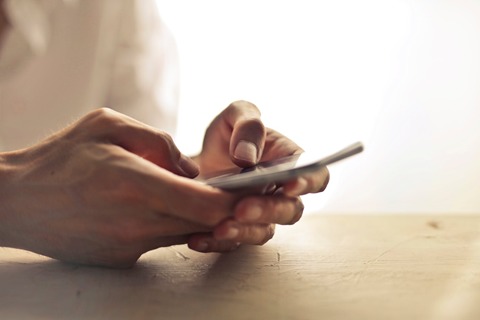
Photo: The proliferation of mobile technology use in low- to middle-income countries offers opportunities for research studies in cognitive ageing and brain health (https://www.pexels.com/).

## MOBILE TESTING: A NEW PARADIGM FOR COGNITIVE TESTING

Without doubt, invaluable lessons have been learnt from years of work and experience acquired conducting research in rich countries. Yet, differences in the context of LIMCS are likely to require innovative approaches for the acquisition of data at a larger and global scale. The implementation of cheaper and less resource demanding means of gathering data in these regions, such as mobile cognitive testing provides opportunities to continue and expand existing studies. Not only are these tests less resource intense, they may also have greater utility than the measures that find favour and are hard to counter in richer countries. However, the success of these developments will necessitate fluid interactions between developers and locally based researchers who are knowledgeable of the local culture and who can aid developers design adequate tools.

Second, the impact of language in cognitive tests in LIMCS is yet to be addressed with many of the more traditional tests being translated into many languages – these have been predominantly for countries involved in clinical trials. However, as differences within languages across regions still exist [16], translations would still need to account for local idioms and language forms, whilst guaranteeing content and difficulty equivalence across versions. Mobile data gathering offers a unique opportunity to implement validation studies quickly and cheaply. Recently, Humphreys et al [17] incorporated a mobile tablet version of the Oxford Cognitive Screen – Plus (OCS-Plus) in a cross-sectional study of a relatively large sample of mid-to-later life adults living in a rural community of South Africa. The OCS-Plus is a domain-specific (language and memory) and domain-general (executive function and attention) measure designed to minimise language and low-literacy confounds associated with traditional tools. The researchers found high task compliance and good validity of the mobile measure, reporting substantive gains in speed and ease of the automated data collection and management features. Longitudinal study is required to assess the validity and feasibility of repeated mobile cognitive assessment, but these initial data do support the use of such technology in large epidemiological studies in low-income countries. Further work to assess the value of mobile cognitive assessment in circumventing other cultural and/or socio-economic confounds associated with traditional cognitive measures is warranted.

The role of other cultural factors, beyond language, on neuropsychological testing has been extensively studied [18]. They include, amongst others, factors such as familiarity with the tests and testing situations, strategies and attitudes to solve tasks, attitude to follow instructions and considerations about privacy [19]. Some of these factors could have a reduced impact on mobile testing batteries by design, such as those related to privacy, interactions with the interviewers and the following of instructions given by interviewers. Even so, there is a move within cross-cultural neuropsychology to bypass many of the cultural and linguistic caveats associated with applying classical cognitive measures across diverse populations, by centering assessments around tasks that have minimal language demands. Visually-based memory tasks which require participants to respond to features of figures/shapes (eg, number, form and/or colour of dots from one trial to another) rather than word lists and story vignettes might be suitable and sensitive alternative measures for detecting cognitive decline in populations with lower literacy and greater language diversity. More recently, some of these tasks have been developed for computerized administration and might be more readily converted to mobile phone-friendly applications. The application of smartphones for cognitive testing of older adults is still in its nascency in wealthier countries. A smartphone version of the Colour-Shape Test, a simple processing speed task that requires test-takers to match shapes with corresponding colours as quickly as possible, has been trialled in a small non-demented sample of American older adults [20]. The results of the study supported the feasibility of using such applications in older adults who had a range of experience with the use of smartphone technologies. The adapted measure also showed good validity, correlating with established measures of global cognition and other measures of processing speed and attention. Given the limited language and cultural confounds presented by this visually-based measure, this application might be readily piloted in low-income country contexts.

Smartphone cognitive testing also offers the opportunity to collect data more often and in a larger number of individuals than in traditional designs, enhancing opportunities to accurately estimate within person change and its onset and to detect associations with possible risk and protective factors [21,22]. Individuals enrolled in these studies could take the tests multiple times over short- and long-term periods of time, could be reminded of engaging in the testing via text messages or other easy to deliver reminders. Hence, researchers would be better placed to understand baseline level of functioning, its short- and long-term variability and change over time. Further, alternative versions of the tests could be easily deployed, and feasibility and exploratory data collections could also be easily implemented. However, the success of these initiatives relies on the quality and availability of the regional mobile network infrastructure. Limited access to power supplies may further impose barriers to smartphone utilisation by more rural communities.

As mobile technologies continue their expansion in LMICs, geographical limitations to access research participants can also be overcome. Mobile phones will ease access to remote or rural communities and the collection of data from populations living in these areas will be facilitated. Importantly, although gender gaps in access to smart technologies exist, the gap is forecasted to be reduced [23], which will in turn help reduce potential gender differences in participation and retention in research studies.

Some other challenges in mobile cognitive testing already present in high income countries and are likely to persist in LMICs. For older participants, issues regarding visual acuity, hearing and dexterity may impede their ability and willingness to perform mobile cognitive exams, particularly where self-reliant assessments are required. Yet, these limitations are very task specific and not universal. For all ages, mobile testing without guidance or supervision from an interviewer might render greater opportunity for distraction during performance that would otherwise be diminished in a laboratory setting. Future work could assess and enhance the usability of testing devices and software applications for naïve populations or incorporate virtual peer models or training that could provide feedback and encouragement following assessment attempts [24]. Needless to say, further validation of mobile assessment tools, in terms of accessibility, usability, longitudinal sampling convenience and language customization is warranted. Another important advantage of using mobile technologies for data gathering is the opportunity to integrate cognitive testing data with other easily collected data, such as accelerometer data, and hence, enhance opportunities to examine individuals from a multidomain perspective.

Researchers from various disciplines, including software developments, clinical and social science researchers and methodologists based at the University of Edinburgh are joining efforts to make the University a hub for global dementia prevention research by facilitating interdisciplinary research, leading the implementation of dementia prevention studies in LMICs and generating opportunities for collaboration with researchers based in LMICs. Their joint expertise, in addition to their large network of international collaborations in wealthy societies and in LMICs, places the University of Edinburgh in a uniquely favourable position to lead these efforts. Just as in rich economies, the opportunities of these novel technologies for research and clinical care infrastructure in LMICs are not fully realized and will require comprehensive study and piloting to assess institutional readiness for implementation. Importantly, they require joint efforts by locally communities, locally based researchers, developers, funders and experienced researchers based in wealthier countries who can share their knowledge and experience.
